# Stable centrosomal roots disentangle to allow interphase centriole independence

**DOI:** 10.1371/journal.pbio.2003998

**Published:** 2018-04-12

**Authors:** Robert Mahen

**Affiliations:** 1 Photonics Group, Department of Physics, Imperial College London, London, United Kingdom; 2 The Medical Research Council Cancer Cell Unit, Hutchison/MRC Research Centre, Cambridge, United Kingdom; Institut Curie, France

## Abstract

The centrosome is a non–membrane-bound cellular compartment consisting of 2 centrioles surrounded by a protein coat termed the pericentriolar material (PCM). Centrioles generally remain physically associated together (a phenomenon called centrosome cohesion), yet how this occurs in the absence of a bounding lipid membrane is unclear. One model posits that pericentriolar fibres formed from rootletin protein directly link centrioles, yet little is known about the structure, biophysical properties, or assembly kinetics of such fibres. Here, I combine live-cell imaging of endogenously tagged rootletin with cell fusion and find previously unrecognised plasticity in centrosome cohesion. Rootletin forms large, diffusionally stable bifurcating fibres, which amass slowly on mature centrioles over many hours from anaphase. Nascent centrioles (procentrioles), in contrast, do not form roots and must be licensed to do so through polo-like kinase 1 (PLK1) activity. Transient separation of roots accompanies centriolar repositioning during the interphase, suggesting that centrioles organize as independent units, each containing discrete roots. Indeed, forced induction of duplicate centriole pairs allows independent reshuffling of individual centrioles between the pairs. Therefore collectively, these findings suggest that progressively nucleated polymers mediate the dynamic association of centrioles as either 1 or 2 interphase centrosomes, with implications for the understanding of how non–membrane-bound organelles self-organise.

## Author summary

Many subcellular organelles are not enclosed by a lipid membrane but instead exist freely in the cellular interior. How the number and position of such non–membrane-bound organelles are maintained is a general unanswered question. Here, I study this problem by focusing on the centrosome—an organelle that nucleates microtubules in fundamental cellular processes. Despite much work characterising the properties of centrosomes, little is known about centrosomal fibres, called roots, which have been suggested to maintain a single centrosome. Using a combination of genome editing, cell fusion, and live-cell imaging, I show that roots are diffusionally stable polymers that assemble slowly over many hours and are able to disentangle as centrosomes transiently split apart into 2 separate units held by the fibres. I propose a model of centrosome number and position regulation using stable fibres, which project outwards into the cytoplasm to form an interface with the surrounding cellular milieu. This model could help our understanding of how organelle position and number are maintained in cells.

## Introduction

The centrosome is a major microtubule organising centre, with critical roles in cell migration, division, shape maintenance, and cilia function. Animal interphase cells are generally thought to have 1 centrosome, consisting of 2 mature microtubule-based structures, called centrioles. Centriole pairs are proteomically and functionally distinct [1] yet apparently remain physically associated, a phenomenon called centrosome cohesion [2–7]. Experimental changes to intercentriolar distance during interphase result in defects in cell migration, ciliary function, and mitosis [8–12], underscoring the functional importance of centrosome cohesion. How 2 centrioles coordinately assemble into a single centrosome yet maintain distinct functions is largely unexplored.

Centrioles are not bounded by a lipid membrane but instead by 2 distinct structures, termed the pericentriolar material (PCM) and pericentriolar fibres [2,13]. Current models of PCM assembly emphasise high dynamics of constituent proteins, potentially as a liquid-like, toroidal structure [14,15]. In contrast, comparatively little is known about either the structure or assembly of pericentriolar fibres. Rootletin, or ciliary rootlet coiled-coil protein (gene symbol *CROCC*), localises to pericentriolar filaments, and rootletin knockout or knockdown results in both loss of filaments and centrosome cohesion [2,8,16,17]. CNAP1, a paralogous gene, interacts with rootletin, likely at centriole proximal ends [2,18]. One model posits that rootletin pericentriolar fibres directly connect centriole pairs to keep them spatially restricted [2,5,16,18]. Consistent with this proposal, rootletin is not found on mitotic centrosomes [5,18–20]. The kinetics of pericentriolar fibre dissolution, when they reform, and the principles governing their replication are poorly understood, however.

To address these questions, this study uses fluorescence imaging, genome editing, and cell fusion to obtain unprecedented spatiotemporal information about the morphology, dynamics, and assembly properties of rootletin fibres, which are referred to as roots. Roots are bifurcating adhesive structures that are licensed to form on centrioles by polo-like kinase 1 (PLK1) enzymatic activity. Both mother and daughter centrioles form independent roots that do not remain connected in response to organelle movement in vivo. Thus, they adopt a structure and function that allow centriole pairs to independently position during interphase, providing new insight into centrosome self-organisation.

## Results

### Centrosomal roots are large bifurcating fibres licensed to form on procentrioles by PLK1 activity

**Fig 1:**
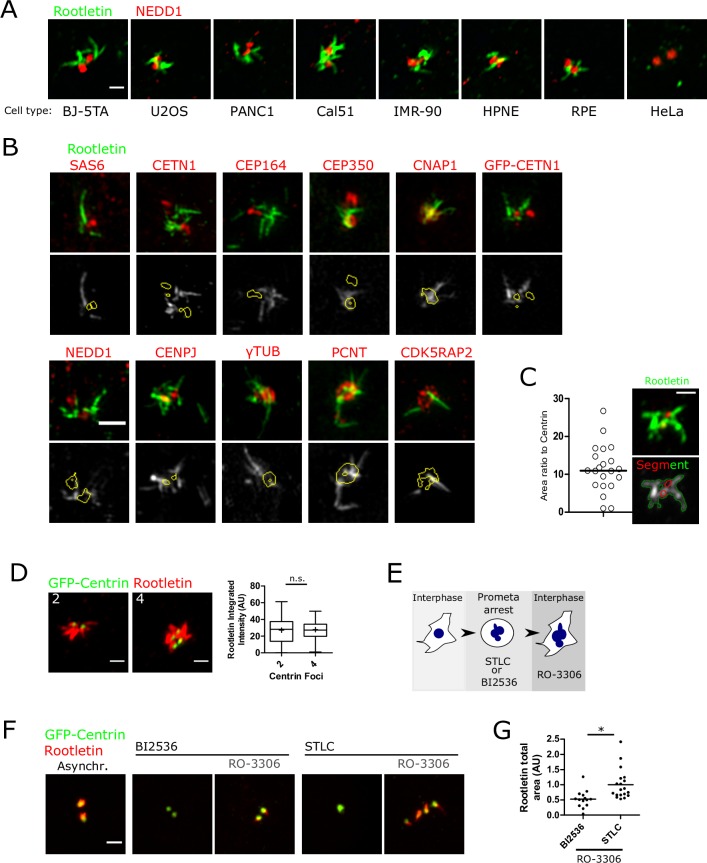
Centrosomal roots are large bifurcating fibres licensed to form on procentrioles by PLK1 activity. (A) Systematic immunofluorescent airyscan imaging of rootletin (green) and the PCM marker NEDD1 (red), using the same conditions throughout all cell types. Confocal slices are shown. Scale bar 1 μm. (B) 3D SIM imaging of rootletin (green) and various centrosomal components (red). Rootletin is stained either by anti-rootletin antibody or by anti-GFP nanobody. Z-projections and single z-slices with segmentation are shown on the top and bottom rows, respectively. Scale bar 1 μm. (C) Quantification of the ratio of rootletin immunostaining area relative to GFP-Centrin 1 area from maximum-intensity projected airyscan images. (D) Rootletin immunofluorescent staining is equal in unreplicated centrosomes and diplosomes. Centrosomes were classified based on GFP-Centrin1 foci number, and anti-rootletin staining was segmented. Scale bar 1 μm. The mean is shown as + and the median as a horizontal bar. n.s., *t* test. *N* = 21 cells. Note that rootletin is shown in red in this panel. (E) Cells were arrested in prometaphase with either STLC (Eg5 inhibition) or BI2536 (PLK1 kinase inhibition) before being forced into interphase with RO-3306 (CDK1 inhibition). (F) Cells expressing GFP-Centrin1 (green) were treated as depicted in panel E before staining with anti-rootletin antibody (red). Maximum-intensity projections are shown. Scale bar 1 μm. (G) Root area per cell was quantified by direct segmentation of rootletin staining from images obtained as described in panel F. The horizontal bar shows the median. *P = 0.0006, *t* test. See S1 Data for source data for the charts. 3D, three-dimensional; CDK1, cyclin-dependent kinase 1; NEDD1, neural precursor cell expressed, developmentally down-regulated 1; PCM, pericentriolar material; PLK1, polo-like kinase 1; SIM, structured illumination microscopy; STLC, S-trityl-L-cysteine.

Pericentriolar filaments near centrosomes have been described for many decades [21], but their ubiquity in different cell types is unknown. The prevalence of rootletin fibres was systematically documented by immunofluorescent staining and enhanced confocal airyscan imaging in a range of human cell types, whether cancerous, immortalised, or primary. Thorough antibody validation, obtained by multiple independent lines of evidence, ensured specific recognition of rootletin (S1 Fig and summarised in Materials and methods). Endogenous rootletin almost ubiquitously formed bifurcating fibres at the centrosome, henceforth referred to as roots (Fig 1A). Costaining and segmentation of a range of markers of either centrioles or the PCM showed limited overlap with roots by either three-dimensional (3D) structured illumination microscopy (SIM) super-resolved imaging (Fig 1B) or confocal airyscan (S1E Fig), indicating that roots occupy a different locale, adjacent to the PCM and centrioles. Segmentation of both roots and centrioles, as marked by a stable green fluorescent protein–Centrin1 fusion (GFP-Centrin1), showed that roots are large relative to centrioles, at approximately 10-fold the size of a centriole on average in retinal pigment epithelium (RPE) cells (Fig 1C). Roots were much shorter than ciliary rootlets—the prominent rootletin fibres found in specialised ciliated cell types, including photoreceptor cells—however ([16,17]; S1F and S1G Fig).

Centriole replication normally proceeds through the appearance of a nascent procentriole from the base of an existing centriole during S and G2 phase [22,23]. To examine whether procentriole formation influences root structure, centrosomes containing either 2 or 4 GFP-Centrin1 marked centrioles were classified, corresponding to either unreplicated centrioles or diplosomes, respectively. No difference in rootletin intensity or size was detected (Fig 1D), suggesting that procentriole growth does not influence root structure.

Procentrioles mature into centrioles during mitosis, dependent on PLK1 activity, becoming replication competent after physically moving away from a centriole (a process termed disengagement) [24]. Therefore, the effects of PLK1 kinase inhibition on root formation were investigated (Fig 1E). Cells arrested in mitosis through PLK1 blockade contained monopolar spindles [25], which were devoid of roots (Fig 1F), consistent with previous work [5,16,18–20]. Because the inhibition of PLK1 results in cell cycle arrest, mitotic exit was forced into an ensuing interphase without cell division—by addition of the cyclin-dependent kinase 1 (CDK1) inhibitor RO-3306 [26]—to understand subsequent effects on root structure in interphase. Control cells were also arrested in mitosis, but instead using the Eg5 kinesin motor inhibitor S-trityl-L-cysteine (STLC) followed by RO-3306. Cells forced into interphase in this manner reformed roots despite unsuccessful mitotic genome segregation (Fig 1F; right-hand panel). However, forced mitotic exit after PLK1 blockade resulted in partial root reformation relative to STLC control (Fig 1G). These results suggest that centrioles are capable of root reformation in G1 regardless of PLK1 activity in the previous mitosis. In contrast, procentrioles must be modified by PLK1-dependent processes before they are competent to form roots in the next cell cycle. Furthermore, because PLK1 promotes centrosomal PCM expansion during mitosis [14], mitotic centrosomes disassemble roots even in the absence of centrosome maturation. Taken together, roots are large bifurcating fibres, found commonly in a range of cell types on mature PLK1-modified centrioles during the interphase.

### Diffusionally stable roots are progressively formed from anaphase

**Fig 2:**
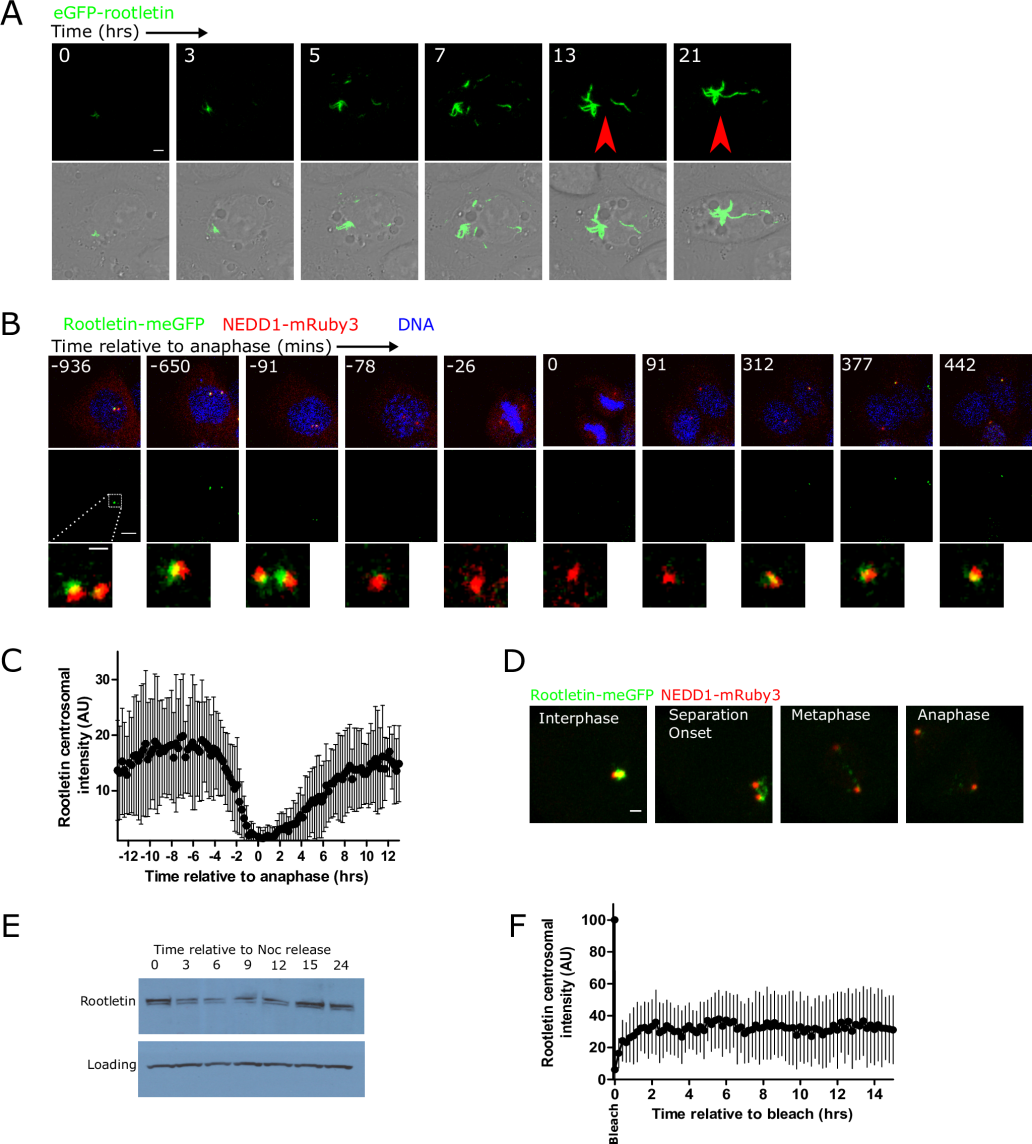
Diffusionally stable roots are progressively formed from anaphase. (A) eGFP-rootletin fibres progressively assemble following transfection. The images are timepoints from a single cell, taken by live-cell 3D confocal time-lapse imaging. The arrows point to a fusion event of 2 preexisting fibres. Scale bar 3 μm. See also S1 Video for the full time course. (B) Representative images from single-cell 3-colour 3D confocal time-lapse imaging of rootletin-meGFP (green), NEDD1-mRuby3 (red; marking the PCM), and DNA (blue; marked by SiR-hoechst), showing root disassembly during mitosis. Images were smoothed for display purposes here using a 2-pixel median filter, but not for analysis. Scale bar 1 μm. See also S2 Video. (C) Cell cycle–dependent changes in rootletin-meGFP centrosomal fluorescence intensity. Centrosomes were automatically tracked as described in Materials and methods. Individual cell traces were manually aligned relative to anaphase onset based on SiR-hoechst staining of DNA (time 0). Mean +/- SD; *N* = 17 cells. (D) Root splitting during centrosome separation in early mitosis, showing rootletin-meGFP (green) and NEDD1-mRuby3 (red). Scale bar 2 μm. (E) Cell cycle–dependent changes in rootletin levels by western blot. Cells were synchronised in nocodazole, released, and harvested at different cell cycle stages, and western blotted with anti-rootletin antibody. (F) FRAP recovery curve over 15 hours, plotting the mean ± SD centrosomal intensity of rootletin-meGFP from 3D confocal imaging after bleaching the fluorescence of the whole centrosome, in thymidine-arrested cells. Centrosome position was tracked independently of rootletin-meGFP fluorescence through simultaneous NEDD1-mRuby3 imaging in a spectrally distinct channel. *N* = 11 cells. FRAP, fluorescence recovery after photobleaching; meGFP, monomeric enhanced green fluorescent protein; NEDD1, neural precursor cell expressed, developmentally down-regulated 1; PCM, pericentriolar material.

The dynamics and biophysical properties of enhanced GFP (eGFP)-tagged rootletin were examined in living cells by utilising both cDNA transgene overexpression and tagging of endogenous alleles. Consistent with previous work [2,16], overexpression of eGFP-rootletin resulted in fibres and bifurcating fork structures that were longer than endogenous rootletin (e.g., compare S2A Fig with Fig 1). Time-lapse imaging of eGFP-rootletin fibre formation following transfection showed that eGFP-rootletin first appeared focally in a single location, prior to the emergence of a larger network over many hours, eventually filling the cytoplasm (Fig 2A and S2A Fig). Fibres increased in size not only by extension in length outwards from a single point but additionally by the coalescence of multiple fibres to form larger aggregates, frequently through end-on fusions (compare arrows in Fig 2A; S1 Video).

Cell cycle–dependent changes in the centrosomal intensity of meGFP-tagged rootletin were followed by 3D confocal time-lapse imaging. Because overexpressed rootletin fibres were larger than endogenous antibody stained roots, and overexpression can influence quantitative measures of protein function in vivo [27], CRISPR Cas9 was used to insert an in-frame fusion of meGFP into the endogenous rootletin (*CROCC*) locus and therefore study rootletin behaviour with live-cell microscopy at endogenous levels for the first time (S3 Fig). Homozygous tagging in the diploid breast cancer cell line Cal51 resulted in fluorescent signal closely resembling antibody staining (S3E Fig). Rootletin-meGFP was barely detectable at the centrosome during mitosis, consistent with immunofluorescent staining (Fig 1), and consequently, a stably coexpressed NEDD1-mRuby3 fluorescent fusion was used to mark the PCM and allow tracking of centrosomes throughout the cell cycle and independently of rootletin levels, in a spectrally distinct fluorescent channel (Fig 2B; S2 Video). Additionally, fluorescently labelled chromatin was monitored to visualise mitotic substages. Rootletin began to be released from the centrosome >2 hours prior to anaphase (Fig 2C). By anaphase, centrosomal rootletin could not be detected above cytoplasmic levels, suggesting disassembly of all centrosomal roots. Rootletin centrosomal levels increased from anaphase but, unexpectedly, continued to increase at a slow rate for approximately 9 hours and thus significantly into G1 phase. Staging of rootletin intensity relative to centrosome separation revealed that its release from the centrosome began prior to centrosome separation and continued after it, with low levels of rootletin still present during centrosome separation, which could be ripped apart during poleward centrosome migration (Fig 2D). Because roots were disassembled in mitosis, it was investigated whether cytoplasmic mitotic rootletin levels were decreased, by western blotting of synchronised cells (Fig 2E). Rootletin cytoplasmic levels were high in mitosis by western blot despite the absence of roots, indicating that cytoplasmic and centrosomal rootletin levels do not always correlate. Together, these results suggest that the removal of rootletin from centrosomes begins early in mitosis or in late G2 phase of the cell cycle, prior to both chromatin condensation and centrosome separation, and then continues during these processes. Rootletin assembly at the centrosome begins from anaphase and occurs slowly for approximately 9 hours into G1 phase.

Previous work has implicated the centriole proximal factor CNAP1 in rootletin centrosomal localisation [2,12,18], and in agreement, small interfering RNA (siRNA)-mediated knockdown of CNAP1 resulted in reduced centrosomal rootletin localisation (S4A Fig). It was investigated whether ectopic CNAP1 plasma membrane localisation via a C-terminal CAAX domain fusion [28] was sufficient to induce root polymerisation outside of the centrosome (S4 Fig). However, neither plasma membrane–localised mScarlet-CNAP1-CAAX, nor the CNAP1-binding partner CEP135 (membrane localised as CEP135-mScarlet-CAAX [29]) induced the formation of ectopic roots.

Some PCM components show dynamic exchange of subunits on the seconds timescale—a property that is thought to be important for centrosome assembly [13]. Fluorescence recovery after photobleaching (FRAP) was therefore used to ask whether rootletin forms steady state polymers. FRAP of extended eGFP-rootletin fibres showed almost no movement of eGFP-rootletin over a time period of 10 minutes, however—even after a relatively rapid bleach (S2B Fig; approximately 1 second). Lack of recovery was not due to image bleaching or fibre movement out of the field of view, because adjacent unbleached ends of the fibre remained unchanged. To investigate very slow dynamic exchange of endogenous centrosomal rootletin-meGFP, on the hours timescale, cells were arrested at the G1/S phase boundary of the cell cycle using thymidine, to circumvent the effects of cell cycle progression on root morphology (Fig 2C). Total centrosomal rootletin-meGFP fluorescent signal was then bleached, and recovery followed by tracking of NEDD1-mRuby3 marked centrosomes during time-lapse imaging (Fig 2F). Recovery of rootletin-meGFP fluorescence on this long timescale was limited to approximately 30%. Together, it can be surmised that eGFP-rootletin fibres are predominantly diffusionally stable structures that are progressively assembled slowly over hours following anaphase.

### Roots disentangle during transient centriole splitting in interphase

**Fig 3:**
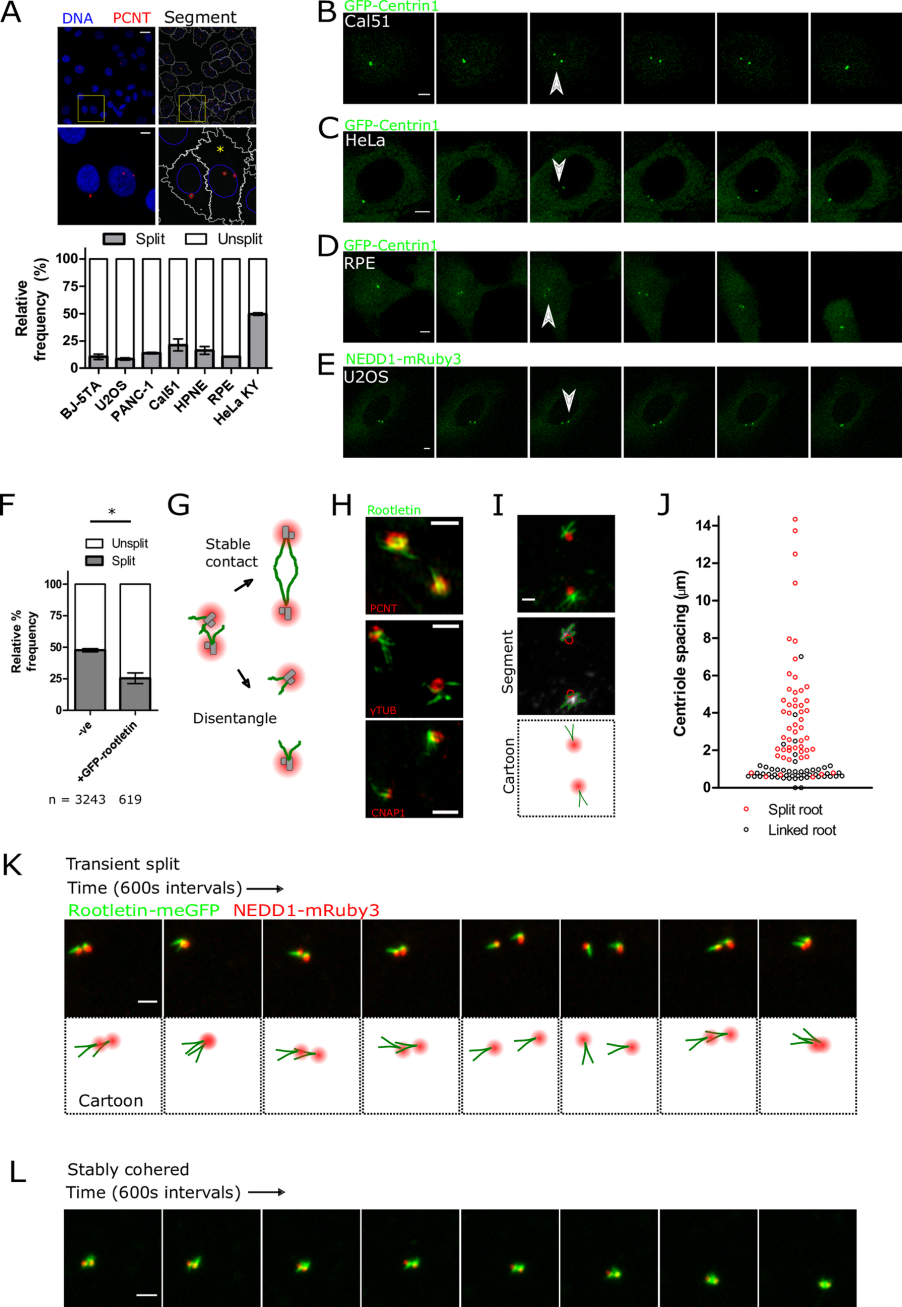
Roots disentangle during transient centriole splitting in interphase. (A) Quantification of centrosome cohesion in the interphase of various cell types through systematic immunofluorescent staining and analysis. The images show representative staining of PCNT (red; marking centrosomal PCM) and DNA (blue; hoechst 44432). The right panel shows representative segmentation of centrosomes (red), nuclei (blue), and cytoplasm (white) in Cal51 cells. The yellow asterisk denotes a cell containing 2 centrosome foci, separated by >1.5 μm. Scale bars 20 μm and 5 μm. The bar graph shows the mean percentage of cells with PCNT centroids separated by >1.5 μm, from a minimum of 500 cells. Error bars show SEM from 2 experiments. (B–E) Selected frames showing centriole splitting in live 3D confocal time-lapse imaging. Centrosomes are marked by either GFP-Centrin1 or NEDD1-mRuby3. Arrows denote centriole splitting events. The time intervals between frames are 12 minutes (panel B and C), 24 minutes (panel D), or 8 minutes (panel E). Scale bar 5 μm. See also S3–S5 Videos. (F) Centrosome cohesion in HeLa cells ± overexpression of eGFP-rootletin, measured by automated imaging and analysis. Horizontal bars show the mean of 2 experiments ± SD. *P < 0.001 by Fischer’s exact test. (G) Opposing models of root behaviour during centriole splitting, termed ‘Stable contact’ or ‘Disentangle’. (H) Representative 3D SIM images of roots (green) after centriole splitting, with the indicated costaining marking either the PCM or centrioles (red). Scale bar 1 μm. (I) Representative airyscan image of roots after centriole splitting. Scale bar 1μm. (J) Root linkage plotted as a function of centriole spacing distance. (K, L) Live-cell airyscan time-lapse imaging of endogenous rootletin-meGFP and NEDD1-mRuby3 during a centriole split (panel K) and when remaining stably cohered (panel L) in Cal51 cells. Scale bar 2 μm. See also S6 Video and S7 Video. See S1 Data for source data for the charts.; meGFP, monomeric enhanced green fluorescent protein; NEDD1, neural precursor cell expressed, developmentally down-regulated 1; PCM, pericentriolar material; PCNT, Pericentrin; SIM, structured illumination microscopy.

How a single interphase cell coordinately organises 2 disengaged centrioles is unclear. The prevalence of centrosomal cohesion was systematically documented in a range of human tissue culture cell types by automated fluorescence imaging and analysis of centrosome position (Fig 3A). Quantification of the percentage of cells with split centrosomes—defined as 2 PCM foci >1.5 μm apart—showed that it was low at approximately 10%, dependent on cell type (see Materials and methods for further discussion of the definition of split centrosome). Thus, in most cell types, centrioles remain cohered in close proximity during interphase, consistent with previous work [6,30–34]. It was investigated whether the minority of split centrioles remain stably separated over time, perhaps due to a permanent failure of centrosome cohesion. However, single-cell 3D confocal live imaging of centriole pairs marked by GFP-Centrin1 showed transient splitting. Therefore, a single mother–daughter centriole pair would split into 2 and then rejoin, often repeatedly (Fig 3B; S3 Video). Transient centriole splitting was manifest in live-cell imaging of several different cell types, including Cal51, HeLa, RPE, and U2OS cells (Fig 3B–3E; S4 Video and S5 Video).

In agreement with a published report [31], HeLa Kyoto cells had high levels of centrosome separation, with approximately 50% of cells showing split centrioles in a fixed asynchronous population (Fig 3A). Because low levels of rootletin expression accompanied short roots in HeLa (Fig 1A) and previous work has shown that rootletin knockdown results in the loss of centrosome cohesion [2,35], the effect of increasing root length by rootletin overexpression on centrosome position was investigated in HeLa cells. This increase in fibre length significantly increased centrosome cohesion in interphase HeLa cells, as measured by automated imaging and analysis of immunofluorescently stained samples (Fig 3F, *P* < 0.001, Fischer’s exact test). Together, these results show that, although mother and daughter centrioles generally remain cohered into a single focal location, they are able to transiently split apart in interphase in a manner that is antagonised by eGFP-rootletin overexpression.

How might rootletin fibres respond to transient centriole splitting? Two opposing models for root behaviour after centriole splitting were postulated (Fig 3G). The first was maintenance of a stable root contact between centrioles as they move apart, for example, due to stretching. The second was loss of physical connection and disentanglement (‘Stable contact’ versus ‘Disentangle’, respectively). Surprisingly and in contrast to cohered centrosomes, rootletin fibres were not detected between split centrioles (Fig 3H, Fig 3I and S5 Fig). Instead, roots from each centriole were generally only detected as linked together at a distance of less than approximately 1.5 μm (Fig 3J), thus supporting the disentanglement model.

Simultaneous 2-colour airyscan microscopy of root disentanglement in living cells revealed that roots occupy markedly heterogenous orientations that change in response to in vivo centriole movement (Fig 3K; S6 Video). The centrosome distal ends of roots have the capacity to pivot relative to centrosome proximal ends, suggesting a common more stable attachment point at the proximal end. Pivoting of centrosome distal tips was not just observed in centrosomes with split centrioles but also in cohered centrosomes, with roots maintained stably at the centriole–centriole interface (Fig 3L; S7 Video). As centrosomes remerged after a split, roots did not necessarily join but could alternatively contact the PCM of the opposing centriole. Together, these observations indicate that although roots can be maintained stably at the interface between mother–daughter centrioles, their orientation is heterogeneous, and notably, in response to centriole movement, a continuous direct rootletin linkage is not detected.

### Independence of mother and daughter centrioles during interphase

**Fig 4:**
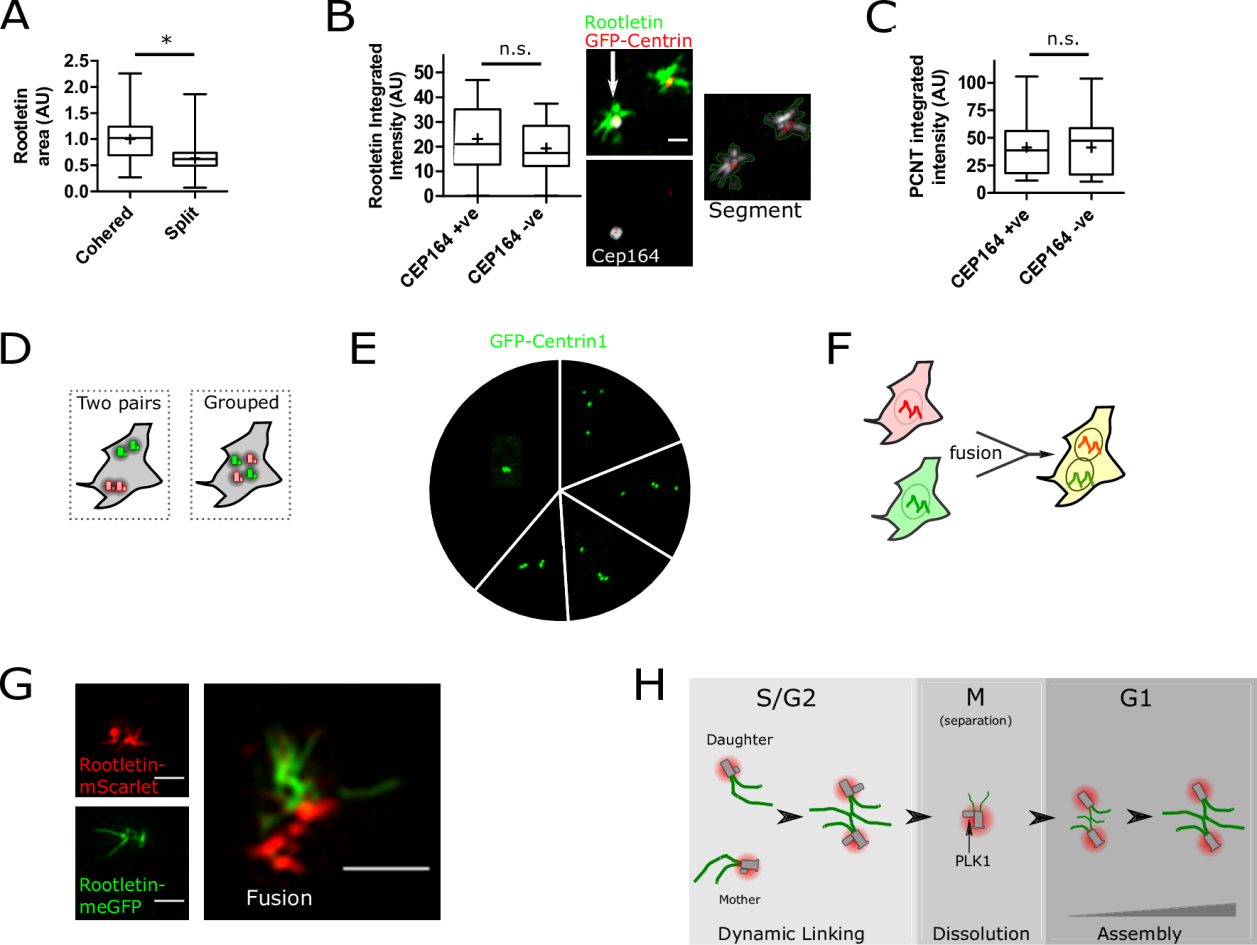
Independence of mother and daughter centrioles during interphase. (A) Root fibre area is significantly lower (*P* < 0.0001, *t* test) in split versus cohered centrioles. Anti-rootletin immunofluorescent staining was imaged and segmented, *N* = 36 cells from 2 experiments. (B) Rootletin immunofluorescent staining (green) is the same at both the mother or daughter centriole (n.s., *t* test). “M” and “D” denote mother and daughter, respectively, on the basis of CEP164 positivity. *N* = 21 cells per sample. Scale bar 1 μm. (C) PCNT immunofluorescent staining (of the PCM) is the same (n.s., *t* test) on either mother or daughter centrioles. Cells were imaged and analysed as described in panel B, except segmenting PCNT. *N* = 21 cells. See S1 Data for source data for the charts. (D) Cells with 4 centrioles might either maintain them as separate pairs or cohere them together (“Grouped”). (E) The pie chart shows the proportion of each GFP-Centrin1 centriole configuration in cells with 4 centrioles, produced as depicted in Fig 1E. The images are representative of each configuration. *N* = 196 cells. (F) Cells expressing endogenously tagged rootletin-meGFP were fused with cells expressing endogenously tagged rootletin-mScarlet. (G) Representative SIM images of single-colour cells and a fused cell, created as depicted in Fig 4F and described in Materials and methods. Scale bars 1 μm throughout. (H) Interphase centriole pairs contain large bifurcating fibres that disentangle when centrioles move apart >1.5 μm relative to each other. Root dissolution begins prior to mitotic centrosome separation and chromosome condensation. At the time of centrosome separation, roots are diminished in quantity and ripped apart during poleward movement of centrosomes. Roots form slowly over many hours from anaphase, as diffusionally stable fibres. PLK1-dependent modification of procentrioles allows root formation in the ensuing cell cycle. meGFP, monomeric enhanced green fluorescent protein; PCNT, Pericentrin; PLK1, polo-like kinase 1; SIM, structured illumination microscopy.

Because disengaged centrioles can transiently split (Fig 3), the comparative structure of roots and PCM on mother and daughter centrioles during splitting was investigated further. Root area was approximately halved in split versus cohered centrioles (Fig 4A), suggestive of equal partitioning of 2 independent roots. Indeed, discrimination of the mother and daughter using CEP164 immunostaining showed that roots are nucleated symmetrically on both mother and daughter (Fig 4B). A similar comparison of PCM structure with the PCM resident Pericentrin (PCNT) showed similarly that both mother and daughter centrioles individually nucleate PCM when split (Fig 4C), something also evident in the live-cell imaging of NEDD1-mRuby3 (Fig 3H–3K) and previous work [24,32,34]. These observations imply that both mature centrioles independently maintain roots and PCM during centrosome splitting in interphase.

Given the dynamic nature of centrosome cohesion (Fig 3) and root disentanglement, it was of interest to investigate whether mother–daughter centriole pairs would be maintained in cells with 4 centrioles (Fig 4D). Centriole position in cells forced into interphase after a failed mitosis by STLC treatment (Fig 1E) showed all possible centrosome cohesion configurations. Most commonly, all 4 centrioles grouped as 1 (Fig 4E), but notably, other spatial arrangements were equally as likely as 2 pairs. Thus, 2 mother–daughter centriole pairs are not maintained separately but will cohere together, even in a grouping such as a single centriole and 3 cohered.

Overexpression of eGFP-rootletin promoted centrosome cohesion in interphase cells with 4 mature centrioles created by sequential STLC/RO-3306 treatment (S6A–S6C Fig), a similar effect to that seen in cells with normal centrosome numbers (Fig 3F). Some cells with supernumerary centrosomes are able to cluster them to form a bipolar spindle during mitosis [36]. It was therefore investigated whether, in contrast to cells with 2 centrosomes (Fig 2), cells with supernumerary centrosomes retained centrosomal rootletin during mitosis. Cells clustering supernumerary centrosomes at spindle poles during mitosis did not contain roots, however (S7 Fig), consistent with a model wherein rootletin does not promote mitotic centriole clustering.

Centrosome cohesion was further examined using polyethylene glycol–mediated cell fusion of 2 different cell lines, 1 expressing endogenously tagged rootletin-meGFP and the other expressing endogenously tagged rootletin-mScarlet (Fig 4F, S3F Fig and see Materials and methods for details of fusion). Fused cells contained centrosomes of 2 fluorescent colours, 1 from each different cell line, as well as 2 nuclei. Because rootletin shows very slow diffusional exchange (Fig 2), this allowed the origin of centriole pairs in fused cells to be distinguished based on the emitted fluorescence. As per after mitotic failure (Fig 4E), mother–daughter centriole pairs were not exclusively maintained after cell fusion, but instead, fluorescent roots of different colours engaged each other (Fig 4G and S6D Fig). Fusion of cell lines expressing rootletin-meGFP or NEDD1-mRuby3 similarly showed that fluorescent roots from 1 cell could embrace all 4 centrioles once fused (S6E Fig). Therefore, by 2 independent methods, mother–daughter centriole pairs are not stably maintained in cells with 4 centrioles.

## Discussion

Cells must carefully regulate centrosome number and position, coordinating 2 centrioles that are capable of distinct functions [23,32,37]. The data here provoke an interesting hypothesis: that interphase cells always have 2 centrosomes that are generally held together by stable fibres that reach outward into the cytoplasm. Three key pieces of evidence are provided. Firstly, both mature interphase centrioles in a pair independently nucleate roots, as well as PCM. Secondly, these units—consisting of a centriole/root/PCM—have the capability to transiently spatially separate during interphase, accompanied by root disentanglement. Thirdly, cells engineered with 2 centriole pairs do not maintain them separately but instead dynamically make new groupings. Thus, there is remarkable pliability in the maintenance of centrosome cohesion, with individual centrioles able to rearrange between pairs, through dynamic splitting of roots. These conclusions are consistent with previous observations of split centrioles [6,30–34]. It is possible that centriole independence may aid plasticity of centrosome function such that the 2 centrioles can either act as one or separately. Thus, the data explain previous observations that centrioles may have either different or coordinated functions [23,32,37]. It cannot be totally excluded that fine rootletin fibres exist, below the detection limit of imaging, which thus keeps centrioles continuously linked. However, there is no evidence for this, either in the motion of roots in living cells or in fixed-cell analyses (Fig 3 and Fig 4), nor is it consistent with previous electron microscopy [2].

How non–membrane-bound organelles regulate their position, size, and number within the cellular interior is still not understood. Recent work has postulated that organelles such as centrosomes and P granules phase-separate as liquid-like compartments [38]. This model is characterised by high internal turnover of components parts, spherical shape, and the ability of multiple organelles to fuse [15]. In contrast, roots are diffusionally stable, remain separate through multiple cycles of merging and splitting, and are not spherical, instead potentially engendering polarity to the centrosome as a branched organelle. Therefore, roots have surprisingly different organisational principles in comparison to the PCM. Further work will be needed to understand whether this has implications for how centrosomal position is regulated. Rootletin loss in mice results in mechanical fragility in ciliated tissues such as photoreceptors, apparently due to the loss of ciliary rootlets [17]. Whether roots contribute to cellular mechanics, in either specialised cell types or nonciliated cycling cells through the maintenance of diffusionally stable contacts, will be an interesting future topic.

In conclusion, root-mediated splitting of 2 centrosomes might allow plasticity of cytoskeletal function, thus explaining how 2 non–membrane-bound organelles coordinately function in either 1 or 2 locations during the interphase [30,34,39]. It is tempting to suggest that progressively nucleated, diffusionally stable polymers might also regulate the subcellular position and number of other organelles.

## Materials and methods

### Antibody validation

Multiple lines of evidence were obtained to indicate that a commercially available anti-rootletin antibody (Novus Biologicals NBP1-80820) specifically recognises the product of the *CROCC* gene. siRNA depletion of rootletin (*CROCC*) using RNA interference removed signal by both immunofluorescence and western blot in multiple cell types (S1A, S1B and S1D Fig). An antibody-independent method—GFP tagging—showed similar protein abundances to measurements made by immunofluorescence, both in time and space (this is apparent throughout Figs 1–3). For example, centrosomal rootletin signal was virtually undetectable in metaphase by either antibody or GFP tagging. Anti-rootletin antibody also stained eGFP-rootletin when overexpressed as a transgene (S1C Fig), and ciliary rootlets in mouse photoreceptor cells (S1F and S1G Fig).

### Cell culture, DNA constructs, and siRNA

Cal51 (German Collection of Microorganisms and Cell Cultures ACC303), U2OS (American Type Culture Collection ATCC HTB-96), HeLa Kyoto, PANC-1, and IMR-90 cell lines were grown in Dulbecco's modified Eagle's Medium (DMEM) supplemented with 10% fetal calf serum, Glutamax, and 100 μg/ml penicillin/streptomycin. hTERT RPE1 cells were cultured in DMEM/F12 with 10% Fetal Bovine Serum (FBS), penicillin/streptomycin, and 4.2% sodium bicarbonate. h-TERT BJ-5ta (ATCC CRL-4001) were grown in a 4:1 mixture of DMEM to M199. h-TERT HPNE (ATCC CRL-4023) were grown in a 3:1 mixture of DMEM to M3:BaseF medium, with 5% fetal calf serum, 10 ng/ml EGF, 2 mM glutamine, and 750 ng/ml puromycin. All tissue culture reagents were purchased from Sigma-Aldrich.

DNA transfection was with lipofectamine 3000 (Invitrogen) according to the manufacturer's instructions. SiR-Hoechst (Tebu Bio) was incubated for 30 minutes at 200 nM before replacing with fresh medium for imaging. siRNA transfection was with RNAiMax transfection reagent (ThermoFisher Scientific). siRNAs used against rootletin (*CROCC*), CEP250/CNAP1, and nontargeting were Dharmacon ON-TARGET plus SMARTpools. siRNA against ODF2 was silencer select from ThermoFisher Scientific (#4427037). NEDD1-mRuby3 contained 5 glycine residues as a linker between the gene and fluorescent protein and was expressed from the vector pcDNA 3.1(+). Plasma membrane targeting was with a C-terminal fusion of the CAAX motif of KRAS4b, consisting of the amino acid sequence KMSKDGKKKKKKSKTKCVIM. mScarlet-cNAP1-CAAX was constructed with HD In-fusion cloning (Clontech), according to the manufacturer’s instructions. CEP135-mScarlet-CAAX was synthesised by GeneArt (ThermoFisher Scientific). Five glycine residues were often, but not always, used as a linker between fusion proteins.

### SIM microscopy

A Zeiss Elyra S.1 equipped with a 63x NA 1.4 lens was used to acquire 16-bit 3D SIM images with 3 rotations and 5 phases. Double-colour labelling was with various combinations of either Alexa 488 or ATTO 488, and either Alexa 594, Alexa 568, or ATTO 565, on cells seeded on high-precision 170-nm glass coverslips (Ibidi μ-slide, 80827). Reconstruction was using Zen Blue software, using automatic parameters. The median (± median absolute deviation) lateral and axial resolution of the system using these settings was measured at 114 ± 4 nm and 352 ± 15 nm, respectively (full-width at half-maximum). Channel alignment was performed in Zen Blue, using a double-colour bead calibration standard.

### cDNA- and CRISPR Cas9–mediated meGFP cell line production

Stable cell lines expressing cDNA constructs were produced by transfection followed by culture for at least 4 days, either with or without antibiotic selection, followed by fluorescence-activated cell sorting. CRISPR clones were produced essentially as described in [27], with some modifications. Guide RNA was expressed from pSpCas9(BB)-2A-GFP (PX458) (Addgene plasmid #48138). Guide RNA sequences all overlapped the *CROCC* STOP codon and against the +ve strand were as follows (5'—3'): CCAGCAGGAGCTCATTTCTC, CCAGAGAAATGAGCTCCTGC, and CAGGAGCTCATTTCTCTGGG. Donor plasmids were constructed in the vector pUC19 by HD In-fusion cloning (Clontech). They consisted of 800 base pair homology arms from the C-terminus of the *CROCC* genomic reference sequence, surrounding the meGFP or mScarlet coding sequence. Five glycine residues linked the gene and fluorescent protein. This insert was cloned into the BamH1 site of the vector pUC19. Insertion of meGFP into the endogenous *CROCC* locus was detected by extraction of genomic DNA using QuickExtract DNA extraction solution (epicentre) according to the manufacturer’s instructions, followed by junction PCR with the following primers: forward: GGCTGGCCTTACCTTCCCTT; reverse: CTGGAAGGCCTGTCACTGTC.

### Immunofluorescence

Tissue culture cells were fixed in 4% paraformaldehyde or ice-cold 100% methanol for 10 minutes, permeabilised in 0.1% Triton, and blocked in 3% bovine serum albumen (ThermoFisher Scientific). Mouse photoreceptor cells were isolated from retina by gentle dissection before fixation and staining as for tissue culture cells. Antibodies used were as follows: rabbit anti-CROCC (1:250–1:750, Novus Biologicals NBP1-80820), mouse anti-NEDD1 (1:500, Abcam ab57336), rabbit anti-PCNT (1:1000; Abcam ab4448), mouse anti-SAS6 (1:300, Santa Cruz Biotechnology sc-81431), mouse anti-CENPJ (1:100, Santa Cruz Biotechnology sc-81432), mouse anti-gamma Tubulin (1:1000, ice-cold methanol; GTU-88), mouse anti-CETN1 (1:4000, EMD Millipore 20H5), mouse anti-CEP164 (1:200, Santa Cruz Biotechnology sc-515403), rabbit anti-CEP350 (1:500, Atlas Antibodies HPA030845), rabbit anti-CNAP1 (Proteintech, 14498-1-AP), rabbit anti-CDK5RAP2 (Atlas Antibodies, HPA046529), alpaca anti-GFP nanobody (1:400, Chromotek gba-488), and FluoTag-X2 anti-mScarlet (1:500, NanoTag Biotechnologies, N1302-At565).

### Image analysis

Images are presented as maximum-intensity projections from 3D data unless otherwise stated. Image brightness and contrast settings were changed linearly and consistently between samples for display purposes of representative images, but not for quantitation. The intensity of centrosomal rootletin-meGFP in cycling cells was determined by automated centrosome tracking after movie acquisition. Centrosomes were segmented and tracked using the Trackmate plugin in ImageJ/Fiji [40], using LAP Tracker, and confirmed as successful by manual analysis of tracking. NEDD1-mRuby3 was tracked, a marker of the PCM that was present throughout the cell cycle. Individual cell tracks were aligned in time relative to anaphase, or the nearest frame to anaphase, based on both bright-field and SiR-hoechst fluorescent DNA labelling.

Segmentation from fixed images was in Cell Profiler software, with data analysis in Knime software. For calculation of per-cell centriole splitting, nuclei were detected based on hoechst staining and cytoplasm by using a watershed algorithm outwards from nuclei based on gamma-tubulin staining. Mitotic cells were excluded based on hoechst staining. Centrosomes were detected with PCNT staining and defined as split if a cell contained 2 PCNT foci centroids >1.5 μm apart by Euclidean straight-line distance. A length of 1.5 μm was chosen as the definition of split centrioles because this distance was the threshold above which roots rarely linked centrioles in imaging (Fig 3J), thus providing an unbiased definition of split centrioles.

For segmentation of roots, various thresholding strategies were used in CellProfiler, including propagation outwards from a GFP-Centrin1 seed region, or direct thresholding. Spacing of PCM staining was measured by adaptive thresholding followed by calculation of 2D Euclidean distance between centroids. Roots were segmented using propagation from PCM and then manually classified as linked if 1 pixel overlap occurred between a root from each PCM. Segmentation of centriole and PCM markers in Fig 1B was in cell profiler using automatic threshold and declumping of adjacent objects based on shape.

### Western blotting

Antibodies used were rabbit anti-CROCC (1:250–1:750 overnight; Novus Biologicals, 80820), rabbit anti-CROCC (1:250–1:750 overnight; Novus Biologicals, 80821), and mouse monoclonal beta-Actin (1:10000 1 hour at room temperature; Sigma-Aldrich). Cells were lysed for 20 minutes on ice in RIPA buffer (50 mM Tris HCl, pH 8, 150 mM NaCl, 1% NP40, 0.5 M sodium deoxycholate, 0.1% SDS, complete protease inhibitor cocktail, PhosSTOP [Roche]). Protein concentration was quantitated using the bicinchoninic acid method (Sigma-Aldrich). Whole-cell extracts were separated by electrophoresis on a 3% to 8% Tris-Acetate gel and transferred to PVDF membrane using the iBlot2 system (ThermoFisher Scientific) according to the manufacturer’s instructions. Membranes were blocked in 5% milk dissolved in 0.1% Tween/TBS.

### Live-cell time-lapse imaging and FRAP

Cells were imaged without phenol red in either L15 CO_2_-indepdendent medium or in Fluorobrite Imaging medium with 5% CO_2_ at 37°C, in Ibidi u-slide 8-well dishes. Imaging was with a Carl Zeiss 880 airyscan, either in airyscan or standard confocal mode, using either a 63x NA 1.4 or 100x NA 1.4 oil immersion lens. Airyscan processing was performed with automatic settings in Zen Black. The median (± median absolute deviation) lateral and axial resolution of the system was measured at 198 ± 7.5 nm and 913 ± 50 nm (full-width at half-maximum), respectively. FRAP was performed essentially as described in [14], bleaching using a 488 argon laser at 100% for the minimum time required to cause approximately 50% fluorescence loss (keeping the same duration in all samples).

### Cell fusion

Cells were fused using Hybri-Max 50% 1450 polyethylene glycol solution (Sigma-Aldrich). Briefly, cells were trypsinised, resuspended in PBS, and mixed at a 1:1 ratio. After spinning, the PBS was aspirated, and PEG was added dropwise over 30 seconds to the cell pellet and left for an additional 3.5 minutes at room temperature. Serum-free medium was then added dropwise for 1 minute before 10-minute incubation at 37°C with normal medium, followed by exchange for fresh medium.

Fused cells constituted approximately 1% of the population and consequently were enriched by fluorescence-activated cell sorting. The fluorescence intensity from endogenously tagged rootletin (either meGFP or mScarlet) was dim as detected by flow cytometry, and so cells were labelled with either CellTrace Violet or CellTrace Far Red dye (ThermoFisher Scientific) to enable efficient sorting. Labelling was for 1 minute at room temperature in PBS, at 500 nM or 20 nM for CellTrace Violet or CellTrace Far Red, respectively. Cells were FACS sorted by gating for either CellTrace Violet, CellTrace Far Red, or NEDD1-mRuby3 positivity relative to negative controls, directly into imaging dishes. The majority of these cells were aneuploid relative to the single-colour lines as expected. Cells with centrosomes marked by NEDD1-mRuby3 fluorescence contained up to 4 foci, due to turnover of this marker at the centrosome.

### Mitotic arrest and release

Cells were arrested for 12 hours in either 200 nM BI2536 (Sigma-Aldrich), 10 μM STLC, or 50 ng/ml Nocodazole. Only mitotically arrested cells were analysed further, by mitotic shake-off. Mitotic exit was forced with RO-3306 (10 μM) for 6 hours, or cells were released from mitotic blockade using 2 washes in warm medium. Dihydrocytochalasin B (DCB) treatment was at 4 μM for 18 hours, followed by 3 washes in fresh medium.

### eGFP-rootletin overexpression in cells with supernumerary centrosomes

Cells were transfected with eGFP-rootletin for 24 hours before overnight arrest in STLC. Mitotic shake-off was performed into RO-3306, allowing a 6.5-hour release. Imaging was by tile-scanning confocal z-stacks. Transfected cells were identified in CellProfiler through segmentation of eGFP-rootletin filaments by global Robust Background threshold. Centrosome cohesion was measured by segmentation of PCNT foci without declumping, thus grouping cohered centrosomes as 1 focus. Split centrosomes were identified in this case as cells with 2, 3, or 4 PCNT foci by Robust Threshold segmentation. Cells either without any detected centrosomes or with greater than 4 foci constituted around 10% of cells, and these were discarded from further analysis.

## Supporting information

S1 Video Growth of cDNA eGFP-rootletin fibres in a single Cal51 cell after transfection. Each frame is taken at a 6-minute interval and shows a maximum-intensity z-projection from a 3D confocal stack. eGFP, enhanced green fluorescent protein.
(AVI)

S2 Video Cell cycle–dependent changes in centrosomal rootletin-meGFP intensity (green; roots) in Cal51cells coexpressing NEDD1-mRuby3 (red; marking the PCM) and stained with SiR-Hoechst (blue; DNA). Each frame is taken at a 12-minute interval and shows a maximum-intensity z-projection from a 3D confocal stack. meGFP, monomeric enhanced green fluorescent protein; NEDD1, neural precursor cell expressed, developmentally down-regulated 1; PCM, pericentriolar material.
(AVI)

S3 Video Centriole splitting and cohesion visualised by 3D confocal time-lapse imaging of GFP-Centrin1 (centrioles) in Cal51 cells. Each frame is taken at a 12-minute interval and shows a maximum-intensity z-projection. Note that this cell divides after 25 frames. Cal51,; GFP, green fluorescent protein.
(AVI)

S4 Video Centriole splitting and cohesion visualised by 3D confocal time-lapse imaging of GFP-Centrin1 (centrioles) in HeLa cells. Each frame is taken at a 12-minute interval and shows a maximum-intensity z-projection. GFP, green fluorescent protein; HeLa.
(AVI)

S5 Video Centriole splitting and cohesion, visualised by 3D confocal time-lapse imaging of GFP-Centrin1 (centrioles) in RPE cells. Each frame is taken at a 24-minute interval and shows a maximum-intensity z-projection. GFP, green fluorescent protein; RPE, retinal pigment epithelium.
(AVI)

S6 Video Root disentanglement during centriole splitting and remerging, visualised by 3D confocal airyscan time-lapse imaging of rootletin-meGFP (green; roots) and NEDD1-mRuby3 (red; PCM). Each frame is taken at a 10-minute interval and shows a maximum-intensity z-projection. meGFP, monomeric enhanced green fluorescent protein; NEDD1, neural precursor cell expressed, developmentally down-regulated 1; PCM, pericentriolar material.
(AVI)

S7 Video Root behaviour in a stably cohered centrosome, visualised by 3D confocal airyscan time-lapse imaging of rootletin-meGFP (green; roots) and NEDD1-mRuby3 (red; PCM). Each frame is taken at a 10-minute interval and shows a maximum-intensity z-projection. meGFP, monomeric enhanced green fluorescent protein; NEDD1, neural precursor cell expressed, developmentally down-regulated 1; PCM, pericentriolar material.
(AVI)

S1 Fig Validation of anti-rootletin antibody (related to Fig 1). (A, B) Anti-rootletin immunofluorescent staining (green) is not evident at centrosomes costained with anti-NEDD1 antibody (red) after rootletin (*CROCC*) siRNA, in multiple cell types. Anti-rootletin staining (green) is present after nontargeting siRNA negative control (nt) treatment. Arrows have been annotated manually to indicate centrosomes. Imaging conditions and brightness and contrast settings are consistent between control and siRNA treated samples. Panel A shows U2OS cells and panel B shows RPE cells. (C) Anti-rootletin antibody immunofluorescently stains (red) eGFP-rootletin overexpressed from a cDNA transgene (green). (D) Anti-rootletin bands are not detected by western blot of whole-cell lysate after rootletin (*CROCC*) siRNA, demonstrating antibody specificity in multiple cell types. (E) Pairwise anti-rootletin antibody costaining of rootletin (green) and other centrosomal genes (red), either in the PCM or centrioles. Maximum-intensity projections of confocal airyscan images; scale bar 1 μm. (F) Anti-rootletin staining (green) in mouse photoreceptor cells. Rootlets extend as part of the inner segment, from nuclei (blue) in the outer nuclear layer. Scale bars 40 μm (left and centre panels) and 20 μm (right panel). (G) Ciliary rootlets (green) costained with anti-NEDD1 antibody in mouse photoreceptor cells. Scale bar 5 μm. eGFP, enhanced green fluorescent protein; NEDD1, neural precursor cell expressed, developmentally down-regulated 1; RPE, retinal pigment epithelium; siRNA, small interfering RNA; nt, nontargeting.
(PDF)

S2 Fig Overexpression of eGFP-rootletin progressively assembles fibres that are diffusionally stable over minutes (related to Fig 2). (A) Representative maximum-intensity z-projection airyscan (i) and SIM (ii) images of overexpressed eGFP-rootletin fibres (green), costained with the PCM marker PCNT (red). Scale bars 5 μm and 1 μm, respectively. (B) FRAP of eGFP-rootletin in the location denoted by the arrow. The graphs show the fluorescence intensity of a line profile along the fibre at each timepoint. Scale bar 1μm. See S1 Data for source data. eGFP, enhanced green fluorescent protein; FRAP, fluorescence recovery after photobleaching; PCM, pericentriolar material; PCNT, Pericentrin; SIM, structured illumination microscopy.
(PDF)

S3 Fig CRISPR Cas9–mediated tagging of endogenous rootletin/CROCC (related to Figs 2 and 3). (A) Schematic of guide RNAs targeting the STOP codon of *CROCC* as well as donor plasmid containing fluorescent protein and homology arms. (B) Clones were screened sequentially by FACS sorting, fluorescence microscopy, and junction PCR. (C) Example overlapping genomic PCR screen of clones expressing rootletin-meGFP. Clone 4_1 was used in this study because it has homozygous tagging of rootletin. Clones 4_7 and 20 are examples of heterozygous and negative clones, respectively. (D) Representative fluorescence microscopy screening of clones expressing endogenous rootletin-meGFP. The bottom panel shows centrosomal fluorescence in positive clones. Scale bar 5 μm. (E) Rootletin-meGFP centrosomal fluorescent signal closely resembles anti-rootletin antibody staining. The image shows clone 4_1 stained with anti-rootletin antibody and imaged by airyscan imaging. Scale bar 1 μm. (F) Overlapping genomic PCR screen of clones expressing rootletin-mScarlet. FACS, fluorescence-activated cell sorting; PCR, polymerase chain reaction.
(PDF)

S4 Fig Ectopic CNAP1/CEP135 localisation to the plasma membrane with a CAAX motif is not sufficient for root formation. (A) siRNA-mediated knockdown of CNAP1 reduces the mean intensity of rootletin immunofluorescent staining at the centrosome. Cells were treated with the indicated siRNA for 18 hours, before immunofluorescent staining with anti-rootletin antibody. Horizontal bars show the mean of the distribution, dots show single cells. nt denotes nontargeting siRNA, -ve denotes untransfected. See S1 Data for source data. (B) Representative 3D SIM image of mScarlet-CNAP1-CAAX (red), costained with anti-rootletin (green) and DNA (Hoechst 44432). The right panel shows a zoomed region of the left panel image. Scale bar 5 μm. Arrows denote plasma membrane. (C) Representative 3D SIM image of CEP135-mScarlet-CAAX (red), costained with anti-rootletin (green) and DNA (Hoechst 44432), as described in panel A. AU, arbitrary unit; nt, nontargeting; SIM, structured illumination microscopy; siRNA, small interfering RNA.
(PDF)

S5 Fig Rootletin links between centriole pairs are not detected using high brightness and contrast settings (related to Fig 3). Rootletin was stained with either anti-rootletin antibody (A) or rootletin-meGFP was stained with anti-GFP nanobody (B) and imaged with 3D SIM. Centriolar PCM was costained with either anti-gamma TUB or anti-PCNT (red). Scale bar 1 μm. meGFP, monomeric enhanced green fluorescent protein; PCM, pericentriolar material; PCNT, Pericentrin; SIM, structured illumination microscopy; g-TUB, tubulin gamma 1 gene.
(PDF)

S6 Fig Centrosome cohesion in cells with supernumerary centrosomes (related to Fig 4). (A) Following transfection with eGFP-rootletin, supernumerary centrosomes were induced as described in Fig 1E, and cells were imaged by confocal tile scanning. Representative immunofluorescent staining (left panel) and segmentation (right panel) of nuclei, PCM, and eGFP-rootletin. Scale bar 5 μm. (B) eGFP-rootletin expressing cells had significantly higher centrosome cohesion relative to untransfected cells. The graph plots the proportion of cells with unsplit (cohered) centrosomes, from the data described in (A). *n* = 778 and 374 cells in -ve and eGFP-rootletin categories respectively. The values are significantly different, p<0.0001, Fischer’s exact test. See S1 Data for source data. (C) The proportion of each configuration of PCNT foci detected from the data described in (A) and (B). (D) Cells expressing endogenously tagged rootletin-meGFP were fused with cells expressing endogenously tagged rootletin-mScarlet and imaged by SIM. Each panel shows a maximum-intensity z-projection of centrosomes in 1 fused cell. Scale bar 1 μm. (E) Cells expressing rootletin-meGFP were fused with cells stably expressing NEDD1-mRuby3. Root arrangement in 3 different fused cells. Scale bar 1 μm. meGFP, monomeric enhanced green fluorescent protein; NEDD1, neural precursor cell expressed, developmentally down-regulated 1; PCM, pericentriolar material; PCNT, Pericentrin.
(PDF)

S7 Fig Cells with supernumerary centrosomes do not retain mitotic roots (related to Fig 4). (A) Supernumerary centrosomes were induced by DCB, which causes cytokinesis failure and tetraploidy. Roots (red) were not detected at centrioles (marked in green by GFP-Centrin1) in mitotic cells, regardless of whether spindles were multipolar (A) or bipolar (B). Image acquisition and presentation settings are constant throughout all 3 panels. Scale bar 5 μm. DCB, Dihydrocytochalasin B; GFP, green fluorescent protein.
(PDF)

S1 Data Raw values from the microscopy data shown in Figs 1, 3, 4, S2, S4 and S6. (XLSX)

## Abbreviations

3D: three-dimensional
CDK1: cyclin-dependent kinase 1
DCB: Dihydrocytochalasin B
DMEM: Dulbecco's modified Eagle's Medium
FBS: fetal bovine serum
FRAP: fluorescence recovery after photobleaching
meGFP: monomeric enhanced green fluorescent protein
NEDD1: neural precursor cell expressed, developmentally down-regulated 1
PCM: pericentriolar material
PCNT: Pericentrin
PLK1: polo-like kinase 1
RPE: retinal pigment epithelium
SIM: structured illumination microscopy
siRNA: small interfering RNA
STLC: S-trityl-L-cysteine
