# Supplementary figures and images for: Stable centrosomal roots disentangle to allow interphase centriole independence
Source: PLoS Biol. 2018 Apr 12;16(4):e2003998. doi: 10.1371/journal.pbio.2003998 (PMC5918241; doi:10.1371/journal.pbio.2003998)

A

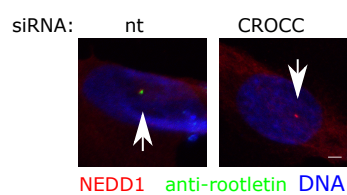

C

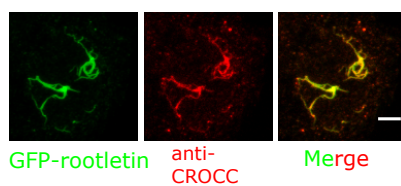

B

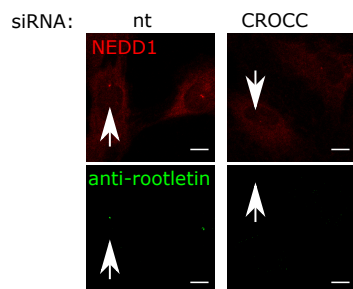

D

Cell type: Cal51 U2OS

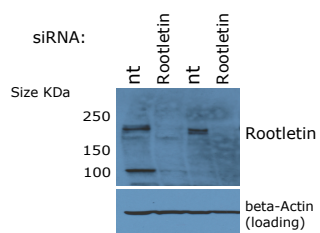

E

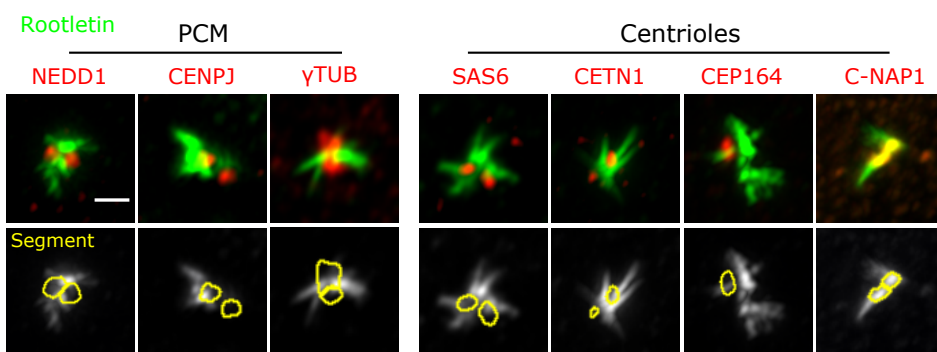

F

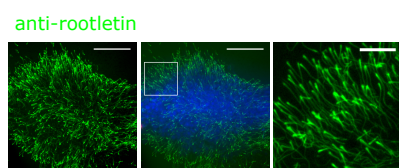

G

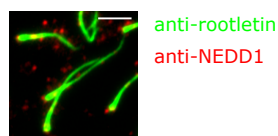

Supplement: S1 Fig — (A, B) Anti-rootletin immunofluorescent staining (green) is not evident at centrosomes costained with anti-NEDD1 antibody (red) after rootletin (CROCC) siRNA, in multiple cell types. Anti-rootletin staining (green) is present after nontargeting siRNA negative control (nt) treatment. Arrows have been annotated manually to indicate centrosomes. Imaging conditions and brightness and contrast settings are consistent between control and siRNA treated samples. Panel A shows U2OS cells and panel B shows RPE cells. (C) Anti-rootletin antibody immunofluorescently stains (red) eGFP-rootletin overexpressed from a cDNA transgene (green). (D) Anti-rootletin bands are not detected by western blot of whole-cell lysate after rootletin (CROCC) siRNA, demonstrating antibody specificity in multiple cell types. (E) Pairwise anti-rootletin antibody costaining of rootletin (green) and other centrosomal genes (red), either in the PCM or centrioles. Maximum-intensity projections of confocal airyscan images; scale bar 1 μm. (F) Anti-rootletin staining (green) in mouse photoreceptor cells. Rootlets extend as part of the inner segment, from nuclei (blue) in the outer nuclear layer. Scale bars 40 μm (left and centre panels) and 20 μm (right panel). (G) Ciliary rootlets (green) costained with anti-NEDD1 antibody in mouse photoreceptor cells. Scale bar 5 μm. eGFP, enhanced green fluorescent protein; NEDD1, neural precursor cell expressed, developmentally down-regulated 1; RPE, retinal pigment epithelium; siRNA, small interfering RNA; nt, nontargeting. (PDF) [file pbio.2003998.s008.pdf]

Ai

PCNT  
eGFP-rootletin

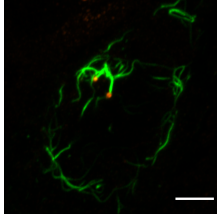

Aii

PCNT  
eGFP-rootletin

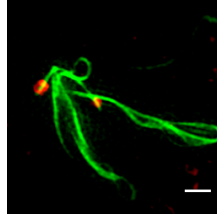

B

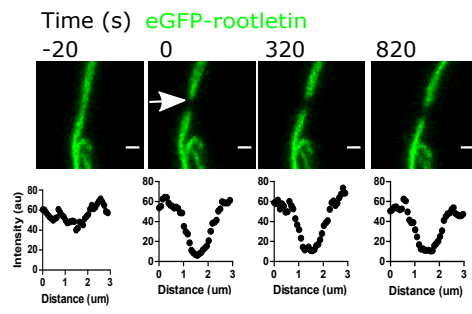

Supplement: S2 Fig — (A) Representative maximum-intensity z-projection airyscan (i) and SIM (ii) images of overexpressed eGFP-rootletin fibres (green), costained with the PCM marker PCNT (red). Scale bars 5 μm and 1 μm, respectively. (B) FRAP of eGFP-rootletin in the location denoted by the arrow. The graphs show the fluorescence intensity of a line profile along the fibre at each timepoint. Scale bar 1μm. See S1 Data for source data. eGFP, enhanced green fluorescent protein; FRAP, fluorescence recovery after photobleaching; PCM, pericentriolar material; PCNT, Pericentrin; SIM, structured illumination microscopy. (PDF) [file pbio.2003998.s009.pdf]

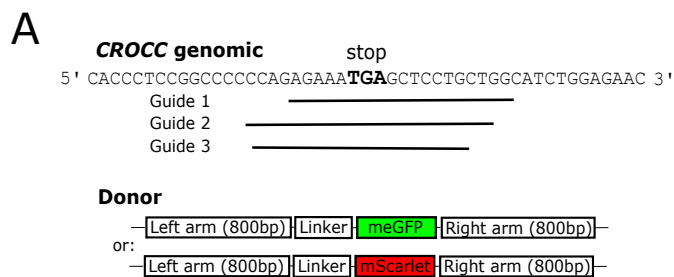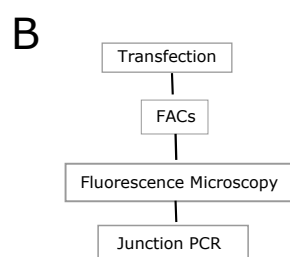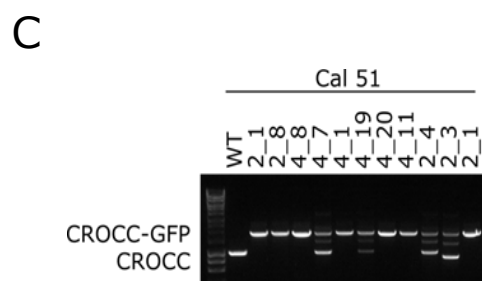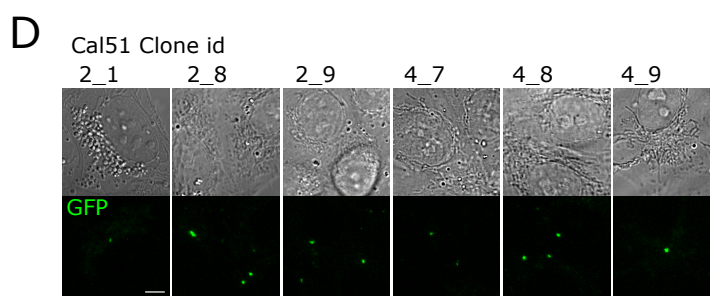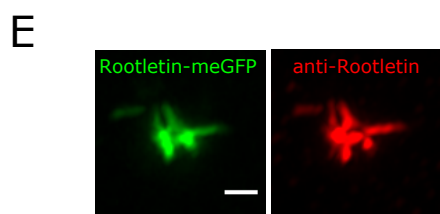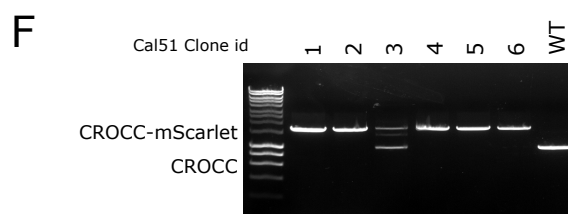

Supplement: S3 Fig — (A) Schematic of guide RNAs targeting the STOP codon of CROCC as well as donor plasmid containing fluorescent protein and homology arms. (B) Clones were screened sequentially by FACS sorting, fluorescence microscopy, and junction PCR. (C) Example overlapping genomic PCR screen of clones expressing rootletin-meGFP. Clone 4_1 was used in this study because it has homozygous tagging of rootletin. Clones 4_7 and 20 are examples of heterozygous and negative clones, respectively. (D) Representative fluorescence microscopy screening of clones expressing endogenous rootletin-meGFP. The bottom panel shows centrosomal fluorescence in positive clones. Scale bar 5 μm. (E) Rootletin-meGFP centrosomal fluorescent signal closely resembles anti-rootletin antibody staining. The image shows clone 4_1 stained with anti-rootletin antibody and imaged by airyscan imaging. Scale bar 1 μm. (F) Overlapping genomic PCR screen of clones expressing rootletin-mScarlet. FACS, fluorescence-activated cell sorting; PCR, polymerase chain reaction. (PDF) [file pbio.2003998.s010.pdf]

**A**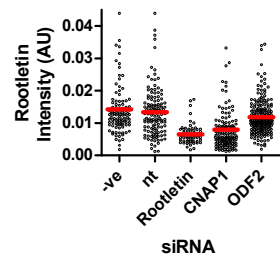**B**

mScarlet-CNAP1-Caax  
Rootletin

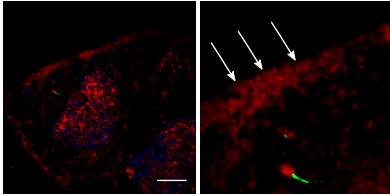**C**

CEP135-mScarlet-Caax  
Rootletin

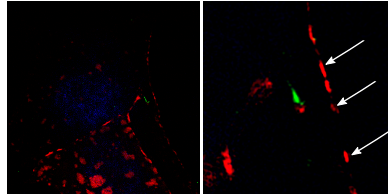

Supplement: S4 Fig — (A) siRNA-mediated knockdown of CNAP1 reduces the mean intensity of rootletin immunofluorescent staining at the centrosome. Cells were treated with the indicated siRNA for 18 hours, before immunofluorescent staining with anti-rootletin antibody. Horizontal bars show the mean of the distribution, dots show single cells. nt denotes nontargeting siRNA, -ve denotes untransfected. See S1 Data for source data. (B) Representative 3D SIM image of mScarlet-CNAP1-CAAX (red), costained with anti-rootletin (green) and DNA (Hoechst 44432). The right panel shows a zoomed region of the left panel image. Scale bar 5 μm. Arrows denote plasma membrane. (C) Representative 3D SIM image of CEP135-mScarlet-CAAX (red), costained with anti-rootletin (green) and DNA (Hoechst 44432), as described in panel A. AU, arbitrary unit; nt, nontargeting; SIM, structured illumination microscopy; siRNA, small interfering RNA. (PDF) [file pbio.2003998.s011.pdf]

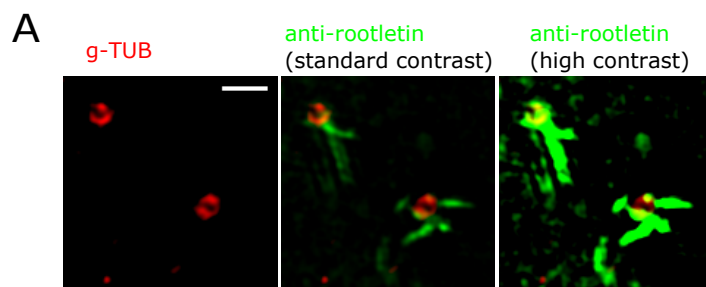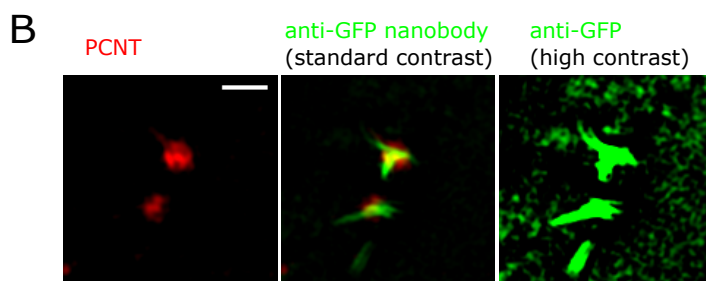

Supplement: S5 Fig — Rootletin was stained with either anti-rootletin antibody (A) or rootletin-meGFP was stained with anti-GFP nanobody (B) and imaged with 3D SIM. Centriolar PCM was costained with either anti-gamma TUB or anti-PCNT (red). Scale bar 1 μm. meGFP, monomeric enhanced green fluorescent protein; PCM, pericentriolar material; PCNT, Pericentrin; SIM, structured illumination microscopy; g-TUB, tubulin gamma 1 gene. (PDF) [file pbio.2003998.s012.pdf]

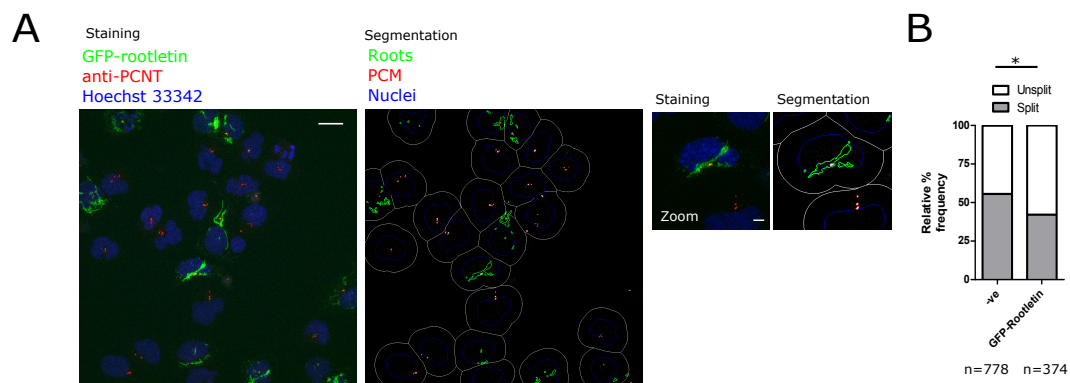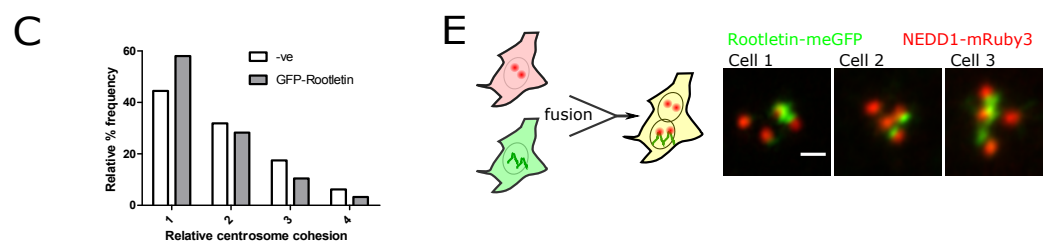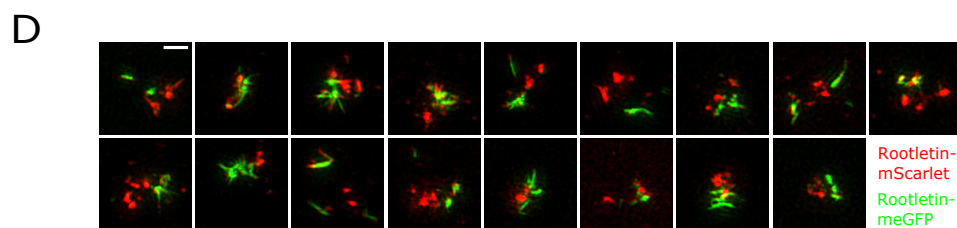

Supplement: S6 Fig — (A) Following transfection with eGFP-rootletin, supernumerary centrosomes were induced as described in Fig 1E, and cells were imaged by confocal tile scanning. Representative immunofluorescent staining (left panel) and segmentation (right panel) of nuclei, PCM, and eGFP-rootletin. Scale bar 5 μm. (B) eGFP-rootletin expressing cells had significantly higher centrosome cohesion relative to untransfected cells. The graph plots the proportion of cells with unsplit (cohered) centrosomes, from the data described in (A). n = 778 and 374 cells in -ve and eGFP-rootletin categories respectively. The values are significantly different, p<0.0001, Fischer’s exact test. See S1 Data for source data. (C) The proportion of each configuration of PCNT foci detected from the data described in (A) and (B). (D) Cells expressing endogenously tagged rootletin-meGFP were fused with cells expressing endogenously tagged rootletin-mScarlet and imaged by SIM. Each panel shows a maximum-intensity z-projection of centrosomes in 1 fused cell. Scale bar 1 μm. (E) Cells expressing rootletin-meGFP were fused with cells stably expressing NEDD1-mRuby3. Root arrangement in 3 different fused cells. Scale bar 1 μm. meGFP, monomeric enhanced green fluorescent protein; NEDD1, neural precursor cell expressed, developmentally down-regulated 1; PCM, pericentriolar material; PCNT, Pericentrin. (PDF) [file pbio.2003998.s013.pdf]

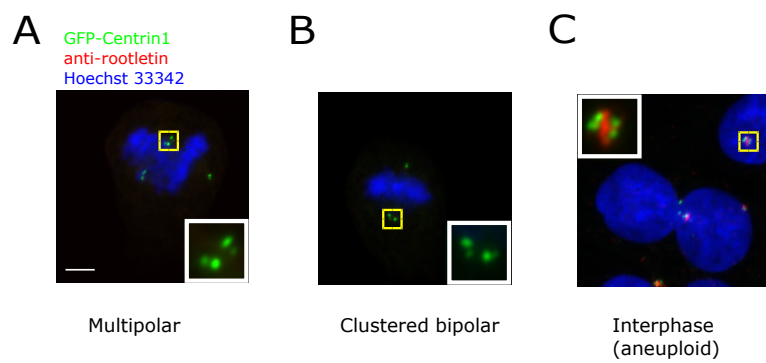

Supplement: S7 Fig — (A) Supernumerary centrosomes were induced by DCB, which causes cytokinesis failure and tetraploidy. Roots (red) were not detected at centrioles (marked in green by GFP-Centrin1) in mitotic cells, regardless of whether spindles were multipolar (A) or bipolar (B). Image acquisition and presentation settings are constant throughout all 3 panels. Scale bar 5 μm. DCB, Dihydrocytochalasin B; GFP, green fluorescent protein. (PDF) [file pbio.2003998.s014.pdf]
